# Event-related potentials and cognitive performance in multiple sclerosis patients with fatigue

**DOI:** 10.1007/s10072-016-2622-x

**Published:** 2016-06-06

**Authors:** Anna Pokryszko-Dragan, Mieszko Zagrajek, Krzysztof Slotwinski, Malgorzata Bilinska, Ewa Gruszka, Ryszard Podemski

**Affiliations:** Department of Neurology, Medical University of Wroclaw, Borowska 213, 50-556 Wroclaw, Poland

**Keywords:** Cognition, Event-related potentials, Fatigue, Multiple sclerosis

## Abstract

The aim of this study was to evaluate event-related potentials (ERP) and cognition in multiple sclerosis (MS) patients with regard to fatigue and disease-related variables. The study comprised 86 MS patients and 40 controls. Fatigue was assessed using the Fatigue Severity Scale (FSS/FSS-5) and the Modified Fatigue Impact Scale (MFIS/MFISmod). N200 and P300 components of auditory ERP were analyzed. Cognition was evaluated by means of Brief Repeatable Battery of Neuropsychological Tests (BRBNT). The results of ERP and BRBNT were compared between non-fatigued, moderately and severely fatigued MS patients and controls. P300 latency was significantly longer in the whole MS group and in the fatigued patients than in the controls. A positive correlation was found between P300 latency and MFIS/MFISmod results, independent from age and MS-related variables. The fatigued patients scored less than non-fatigued ones in tests evaluating memory, visuomotor abilities and attention. Results of these tests correlated significantly with fatigue measures, independently from MS-related variables. Fatigue in MS patients showed significant relationships with impairment within the memory and attention domains. Parameters of auditory ERP, as electrophysiological biomarkers of cognitive performance, were not independently linked to fatigue.

## Introduction

Fatigue is a common complaint among the patients with multiple sclerosis (MS), which essentially affects their condition and daily activities. The origin of fatigue has not been fully elucidated. Its main subjective character gives rise to many controversies and makes the assessment of fatigue especially difficult. In our previous study on that subject [1], we found significant abnormalities in parameters of visual and brainstem auditory evoked potentials in MS patients with moderate and severe fatigue, which might indicate dysfunction of neuronal pathways as the background of fatigue. In further investigation, we aimed to analyze in the same group of patients possible relationships between fatigue and cognitive impairment, another non-physical aspect of MS-related disability receiving increasing attention in recent years. These relationships have already been studied but still remain a matter of debate [2–5]. In the assessment of cognition in MS patients, complementary to the neuropsychological tests, event-related potentials (ERP) have been used, with parameters of N200 and P300 components regarded as an electrophysiological index of cognitive functions [6–13]. Apart from individual reports [14, 15] ERP parameters have not been investigated in the view of fatigue in the course of MS.

The purpose of this study was therefore to evaluate both ERP and cognitive performance in MS patients and to analyze their relationships with the level of fatigue, considering also the impact of clinical disease-related variables.

## Materials and methods

The studied group, described in the previous report [1], involved 86 patients (24 men and 62 women, aged 19–60 years, mean 39.55) diagnosed with clinically definite MS according to McDonald’s criteria [16]. The patients were under the charge of the outpatient MS clinic, Department of Neurology, Medical University of Wroclaw. Seven patients were recognized with clinically isolated syndrome (CIS), 61 with relapsing-remitting, and 18 with secondary progressive MS. The exclusion criteria involved: concomitant diseases known to affect fatigue and/or cognitive functions, current immunosuppressive treatment (a wash-out period of 4 weeks was required from treatment with corticosteroids due to recent relapse) or immunomodulating therapy (62 patients had never been treated with disease-modifying agents, in 24 subjects the treatment was ceased at least 6 months prior to their inclusion in this study).

The control group of 40 healthy volunteers was involved in the study, matched for age, gender and educational level to the MS patients (12 men, 28 women, aged 23–60 years, mean 38.8).

All the subjects gave their informed consent to participate in the study and the project was approved by the Bioethical Committee at the Medical University of Wroclaw.

Symptoms and signs of neurological deficit and level of disability were established on the basis of neurological examination, with the use of Expanded Disability Status Scale (EDSS) [17]. The duration of the disease was determined on the basis of medical records, which allowed to calculate the index of disability progression (Multiple Sclerosis Severity Scale—MSSS) [18].

### Assessment of fatigue and cognitive functions

The assessment of fatigue in MS patients was performed with the use of self-assessment questionnaires: Fatigue Severity Scale (FSS) [19] and Modified Fatigue Impact Scale (MFIS) [20], with their results re-evaluated according to the version of Mills et al. [21, 22], involving the Rasch analysis (FSS-5, MFISmod, respectively). In addition, the MFISmod results were divided into scores from the physical and cognitive subscales of fatigue. As described in the previous study [1], the patients were divided into three subgroups, based on the results of FSS/FSS-5: subgroup I without fatigue (FSS/FSS-5 <3.5), subgroup II with moderate fatigue (FSS/FSS-5 = 3.5–5.5) and subgroup III with severe fatigue (FSS/FSS-5 >5.5).

The Brief Repeatable Battery of Neuropsychological Tests (BRBNT) [23], including: the Selective Verbal Reminding Test (SVRT), Spatial Recall Test (SpaRT), Symbol Digit Modalities Test (SDMT), Paced Auditory Serial Addition Test (PASAT) and Word Generation List (verbal fluency—VF) was administered by a psychologist to evaluate the patients’ cognitive functions. The results of individual tests were referred to normative values [24].

### Event-related potentials

Auditory ERPs were performed with the use of tones of 70 dB intensity and 200 ms duration, presented binaurally via the earphones. The “oddball paradigm” was applied, with target stimuli (high frequency tones: 2 kHz) randomly interspersed among non-target ones (low frequency tones: 1 kHz). The target tones occurred 20 % during the time of each trial and non-target tones 80 % of the time. The subjects were lying in a semi-darkened room, were asked to stay awake and to count mentally the target stimuli. ERPs were recorded in Fz, Cz and Pz (the 10–20 system), referenced to linked earlobes and with a forearm ground. Ag/AgCl surface electrodes were used with their impedance maintained below 5 k Ohm. The responses were recorded and averaged by a Nicolet 1000 Viking, with a 0.30/s, 70 Hz bandpass filter, a sweep time of 1000 ms and a pre-stimulus baseline of 250 ms, with automatic artifact rejection. Two runs were performed for every subject with at least 30 target trials averaged in each run. The records were inspected visually (with additional correction for artifacts) and P300 (the positive component with a latency of 300–500 ms after the start of the stimulus) and N200 (the negative component with a latency of 180–300 ms) were identified. The latencies and amplitudes (measured as “peak to baseline”) of P300 and N200 components were determined [12].

All the procedures were performed on the same day, in morning hours. Considering fatigue as the main subject of evaluation, the sessions including performance of VEP and BAEP [1] were conducted on another day.

### Statistical analysis

Mean and median values with standard deviations were calculated for all the analyzed variables. The data obtained from the whole group of MS patients and subgroups I, II and III were compared with those from the controls, and the results were also compared between the subgroups I, II and III of one-way analysis of variance (ANOVA), alternatively using the non-parametrical Kruskal–Wallis test, when the variances in groups were not homogeneous (the homogeneity of variance was determinate by the Bartlett’s test). Statistical significance between frequencies (gender) was calculated by the Chi-square test with Yate’s correction.

Correlations were searched for between fatigue measures (FSS/FSS-5, MFIS/MFISmod) and ERP parameters, BRBNT results as well as MS-related variables, with the use of Pearson’s correlation coefficient. Multiple regression analysis was used to check the independence of correlations between fatigue measures and ERP and BRBNT results, as well as to check the impact of age and MS-related variables upon the correlations between fatigue measures between ERP and BRBNT, respectively.

*P* < 0.05 was regarded as statistically significant. The statistical analysis was performed using EPIINFO Ver. 3.5.2 software.

## Results

As described in the previous study [1], 29 patients (8 men, 21 women) were assigned as non-fatigued (subgroup I), 31 patients (7 men, 24 women) as moderately fatigued (subgroup II), and 26 (8 men, 18 women) as severely fatigued (subgroup III), considering FSS/FSS-5 score. There were no significant differences in age and gender between these subgroups, or between each of them and healthy controls (Table 1).

**Table 1:** Demographic and disease-related variables in the healthy controls, the whole group of MS patients and subgroups of non-fatigued (I), moderately fatigued (II) and severely fatigued (III) ones

	Control group (*n* = 40)	MS patients (*n* = 86)		Subgroup I (*n* = 29)		Subgroup II (*n* = 31)		Subgroup III (*n* = 26)	
Age (years)									
Range	23–60	19–60	*p* (MS-contr) = 0.64	22–55	*p* (I–contr) = 0.46	19–56	*p* (II-contr) = 0.40	24–59	*p* (III-contr) = 0.48 p (I–III) = 0.2 p (II–III) = 0.95
Mean	38.8	39.5		37.1		40.9	p (I–II) = 0.2	40.7	
Gender									
M	12	24	*p* (MS-contr) = 0.63	8	*p* (I–contr) = 0.78	7	*p* (II-contr) = 0.67	8	*p* (III-contr) = 0.72 p (I–III) = 0.97 p (II–III) = 0.69
F	28	62		21		24	p (I–II) = 0.88	18	
MS duration (years)									
Range		1–30		1–12		0.5–30	*p* (I–II) = 0.09	1.5–30	***p*** **(I–III)** = **0.003** *p* (II–III) = 0.24
Mean		8.57		5.38		9.05		11.56	
SD		8.4		3.41		7.38		8.7	
EDSS									
Range		1–6.5		1–6.5		1–6.5	***p*** **(I–II)** = **0.027**	1.5–6.5	***p*** **(I–III)** = **0.000001** ***p*** **(II–III)** = **0.01**
Mean		3.03		2.12		2.98		4.08	
SD		1.42		1.24		1.66		1.39	
MSSS									
Range		1.1–8.8		1.1–7.9		1.1–8.3	*p* (I–II) = 0.58	1.1–8.8	***p*** **(I–III)** = **0.014** ***p*** **(II–III)** = **0.047**
Mean		4.4		3.86		4.16		5.32	
SD		2.15		2.03		2.06		2.24	

Bold values indicate *p* < 0.05 which are statistically significant

*EDSS* Expanded Disability Status Scale, *MSSS* MS Severity Scale, *SD* standard deviation

In the whole group of MS patients, the results of MFIS ranged from 4 to 64 (mean 36.3), MFISmod: 3–43 (mean 23.2). Mean MFIS scores for subgroups I, II and III were: 21.7, 38.2, 50.5 and in MFISmod: 13.2, 24.4 and 32.9, respectively. MFIS and MFISmod results correlated significantly with FSS/FSS-5 score (*R* = 0.86, *p* = 0.00001 and *R* = 0.85, *p* = 0.00001, respectively). A significant correlation was found between MFIS and MFISmod results and the age of the MS patients (*R* = 0.24, *p* = 0.02 and *R* = 0.21, *p* = 0.04, respectively). There were no such correlations for FSS/FSS-5 results.

The duration of MS in the patients was 1–30 years (mean 8.57), EDSS 1–6.5 (mean 3.03) and MSSS 1.1–8.8 (mean 4.4). The values of these parameters and their comparison for the subgroups I, II and III are presented in Table 1. In the subgroup I, there were 2 patients with CIS, 26 with RRMS and 1 with SPMS, in subgroup II: 5 with CIS, 18 with RRMS and 8 with SPMS, in subgroup III 17 patients with RRMS and 9 with SPMS. Comparing the proportions of particular MS types in these subgroups, significant difference was found between subgroup I and III (*p* = 0.007), but not between I and II (*p* = 0.068) or II and III (*p* = 0.106).

Mean values for N100 latency did not differ significantly between MS patients (as a whole group and in each of subgroups) and the controls or between the subgroups I, II and III. Mean values for N100 amplitude in Pz were significantly lower in all MS patients, subgroup I and III in comparison with the controls, and in CZ—significantly lower in subgroup I in comparison with the controls and subgroup III (Table 2).

**Table 2:** N100 ERP component parameters in the healthy controls, the whole MS group and the subgroups of non-fatigued (I), moderately fatigued (II) and severely fatigued (III) MS patients

	Controls (*n* = 40)	MS patients (*n* = 86)		Subgroup I (*n* = 29)		Subgroup II (*n* = 31)		Subgroup III (*n* = 26)	
Lat.N100 Fz (ms)									
Mean	96.2	96.9	*p* (MS-contr) = 0.47	96.3	*p* (I-contr) = 0.51	97.4	*p* (II-contr) = 0.13 *p* (I–II) = 0.18	96.6	*p* (III-contr) = 0.10 *p* (I–III) = 0.16 *p* (II–III) = 0.21
SD	19.1	19.7		19.4		19.3		20.1	
Lat. N100 Cz (ms)									
Mean	96.5	97.4	*p* (MS-contr) = 0.42	96.8	*p* (I-contr) = 0.54	97.9	*p* (II-contr) = 0.12 *p* (I–II) = 0.21	97.4	*p* (III-contr) = 0.14 *p* (I–III) = 0.22 *p* (II–III) = 0.36
SD	18.5	19.0		18.7		18.9		19.3	
Lat. N100 Pz (ms)									
Mean	97.1	97.5	*p* (MS-contr) = 0.56	97.2	*p* (I-contr) = 0.48	98.2	*p* (II-contr) = 0.19 *p* (I–II) = 0.15	97.5	*p* (III-contr) = 0.41 *p* (I–III) = 0.46 *p* (II–III) = 0.28
SD	18.8	19.2		19.0		19.4		19.6	
Ampl N100 Fz (µV)									
Mean	9.13	8.11	*p* (MS-contr) = 0.19	6.90	***p*** **(I-contr)** = **0.035**	8.31	*p* (II-contr) = 0.21	8.92	*p* (III-contr) = 0.46 ***p*** **(I–III)** = **0.031** *p* (II–III) = 0.17
SD	4.02	3.51		3.23		4.22	*p* (I–II) = 0.09	3.51	
Ampl N100Cz (µV)									
Mean	9.26	7.55	*p* (MS-contr) = 0.08	6.76	*p* (I-contr) = 0.07	7.82	*p* (II-contr) = 0.08 *p* (I–II) = 0.11	8.23	*p* (III-contr) = 0.19 *p* (I–III) = 0.06 *p* (II–III) = 0.38
SD	4.07	3.53		3.13		4.36		3.49	
Ampl N100 Pz (µV)									
Mean	7.34	5.44	***p*** **(MS-contr)** = **0.04**	4.96	***p*** **(I-contr)** = **0.026**	5.64	*p* (II-contr) = 0.06 *p* (I–II) = 0.10	5.14	***p*** **(III-contr)** = **0.032** *p* (I–III) = 0.54 *p* (II–III) = 0. 47
SD	3.62	2.51		2.43		2.75		2.88	

Bold values indicate *p* < 0.05 which are statistically significant

Mean values for N200 latency were significantly longer in all MS patients in comparison with the controls and in subgroups II and III in comparison with the controls. No differences were found between subgroups I, II and III. The studied groups and subgroups did not differ significantly in terms of the values for N200 amplitude (Table 3). Mean values for P300 latency were significantly longer in all MS patients in comparison with the controls and in subgroups II and III in comparison with the controls. There were no differences between subgroups I, II and III. Mean values for P300 amplitude were significantly lower in subgroup III than in I, but only in Pz and (on the border of significance) in Cz references. No other differences were found in the amplitudes of P300 (Table 4).

**Table 3:** N200 ERP component parameters in the healthy controls, the whole MS group and the subgroups of non-fatigued (I), moderately fatigued (II) and severely fatigued (III) MS patients

	Controls (*n* = 40)	MS patients (*n* = 86)		Subgroup I (*n* = 29)		Subgroup II (*n* = 31)		Subgroup III (*n* = 26)	
Lat.N200 Fz (ms)									
Mean	209.5	223.8	***p*** **(MS-contr) = 0.003**	218.8	*p* (I-contr) = 0.11	225.8	***p*** **(II-contr)** = **0.013** *p* (I–II) = 0.33	227.1	***p*** **(III-contr)** = **0.003** *p* (I–III) = 0.17 *p* (II–III) = 0.85
Median	205.0	220.5		220.0		218.0		225.5	
SD	23.1	25.7		23.9		30.6		20.7	
Lat. N200 Cz (ms)									
Mean	207.9	222.3	***p*** **(MS-contr) = 0.004**	217.1	*p* (I-contr) = 0.12	224.7	***p*** **(II-contr)** = **0.012** *p* (I–II) = 0.31	225.3	***p*** **(III-contr)** = **0.003** *p* (I–III) = 0.20 *p* (II–III) = 0.93
Median	204.0	219.0		218.0		217.0		224.0	
SD	22.5	26.9		24.9		31.8		22.3	
Lat. N200 Pz (ms)									
Mean	208.6	222.1	***p*** **(MS-contr) = 0.008**	217.9	*p* (I-contr) = 0.11	223.8	***p*** **(II-contr)** = **0.023** *p* (I–II) = 0.43	224.8	***p*** **(III-contr)** = **0.006** *p* (I–III) = 0.29 *p* (II–III) = 0.89
Median	205.0	220.0		221.0		217.0		224.5	
SD	22.0	27.2		25.3		32.4		22.6	
Ampl N200 Fz (µV)									
Mean	4.27	4.89	*p* (MS-contr) = 0.29	4.64	*p* (I-contr) = 0.59	5.25	*p* (II-contr) = 0.23 *p* (I–II) = 0.49	4.74	*p* (III-contr) = 0.51 *p* (I–III) = 0.89 *p* (II–III) = 0.57
Median	3.80	5.20		4.10		5.40		5.20	
SD	2.91	3.15		2.77		3.82		2.71	
Ampl N200Cz (µV)									
Mean	3.51	4.12	*p* (MS-contr) = 0.31	4.31	*p* (I-contr) = 0.29	4.0	*p* (II-contr) = 0.53 *p* (I–II) = 0.70	4.06	*p* (III-contr) = 0.49 *p* (I–III) = 0.76 *p* (II–III) = 0.94
Median	2.10	3.40		3.70		3.60		3.20	
SD	3.09	3.13		3.03		3.33		3.13	
Ampl N200 Pz (µV)									
Mean	2.69	2.96	*p* (MS-contr) = 0.55	2.96	*p* (I-contr) = 0.67	2.79	*p* (II-contr) = 0.86 *p* (I–II) = 0.6	3.18	*p* (III-contr) = 0.46 *p* (I–III) = 0.74 *p* (II–III) = 0. 49
Median	1.80	2.40		2.20		2.60		2.40	
SD	2.58	2.21		2.36		1.74		2.57	

Bold values indicate *p* < 0.05 which are statistically significant

**Table 4:** P300 ERP component in healthy controls, the whole MS group and the subgroups of non-fatigued (I), moderately fatigued (II) and severely fatigued (III) MS patients

	Controls (*n* = 40)	MS patients (*n* = 86)		Subgroup I (*n* = 29)		Subgroup II (*n* = 31)		Subgroup III (*n* = 26)	
Lat.P300 Fz (ms)									
Mean	321.4	339.4	***p*** **(MS-contr) = 0.0008**	334.4	*p* (I-contr) = 0.10	341.3	***p*** **(II-contr)** = **0.001** *p* (I–II) = 0.39	342.6	***p*** **(III-contr)** = **0.0009** *p* (I–III) = 0.33 *p* (II–III) = 0.86
Median	323.0	341.0		338.0		340.0		344.0	
SD	22.4	29.5		34.0		27.5		26.6	
Lat. P300 Cz (ms)									
Mean	322.6	340.6	***p*** **(MS-contr) = 0.001**	335.8	*p* (I-contr) = 0.054	341.6	***p*** **(II-contr)** = **0.004** *p* (I–II) = 0.47	344.5	***p*** **(III-contr)** = **0.001** *p* (I–III) = 0.29 *p* (II–III) = 0.71
Median	326.0	340.5		338.0		339.0		345.5	
SD	23.4	30.0		32.2		30.0		27.7	
Lat. P300 Pz (ms)									
Mean	325.6	343.1	***p*** **(MS-contr) = 0.002**	338.7	*p* (I-contr) = 0.054	343.9	***p*** **(II-contr)** = **0.005** *p* (I–II) = 0.52	347.0	***p*** **(III-contr)** = **0.002** *p* (I–III) = 0.31 *p* (II–III) = 0.69
Median	328.0	343.0		339.0		342.0		347.0	
SD	23.7	30.0		31.5		30.3		28.5	
Ampl P300 Fz (µV)									
Mean	7.08	6.80	*p* (MS-contr) = 0.72	7.49	*p* (I-contr) = 0.69	6.92	*p* (II-contr) = 0.87 *p* (I–II) = 0.59	5.9	*p* (III-contr) = 0.25 *p* (I–III) = 0.12 *p* (II–III) = 0.34
Median	6.40	6.65		8.50		6.70		6.15	
SD	4.41	3.95		3.93		4.35		3.42	
Ampl P300 Cz (µV)									
Mean	8.10	7.60	*p* (MS-contr) = 0.52	8.47	*p* (I-contr) = 0.72	7.69	*p* (II-contr) = 0.69 *p* (I–II) = 0.46	6.54	*p* (III-contr) = 0.13 *p* (I–III) = 0.052 *p* (II–III) = 0.28
Median	8.20	7.60		8.50		7.40		6.95	
SD	4.32	3.90		3.76		4.32		3.4	
Ampl P300 Pz (µV)									
Mean	8.47	8.33	*p* (MS-contr) = 0.86	9.13	*p* (I-contr) = 0.49	8.73	*p* (II-contr) = 0.80 *p* (I–II) = 0.71	6.96	*p* (III-contr) = 0.13 ***p*** **(I–III)** = **0.029** *p* (II–III) = 0.11
Median	7.60	8.25		8.80		8.30		7.20	
SD	4.09	4.0		3.67		4.52		3.46	

Bold values indicate *p* < 0.05 which are statistically significant

**Fig. 1:**
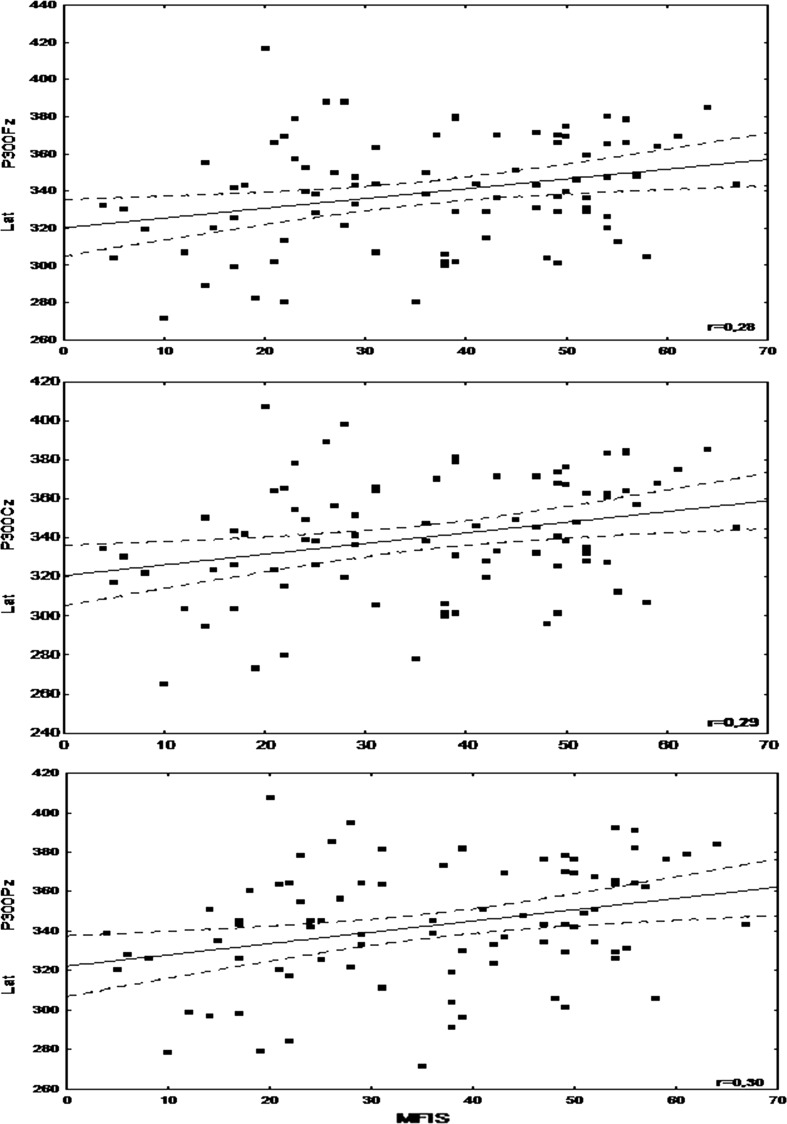
Correlation between P300 latency and Modified Fatigue Impact Scale (MFIS) results

No correlations were found between N200 parameters and fatigue measures (FSS/FSS-5 and MFIS/MFISmod) or the duration of MS, but N200 latency showed a significant positive correlation with EDSS (*R* = 0.24, *p* = 0.02) and MSSS (*R* = 0.22, *p* = 0.04). P300 latency showed a significant positive correlation with the results of MFIS, MFISmod and the physical subscale of MFISmod (Table 5; Fig. 1). Multiple regression analysis showed that these correlations were independent from the impact of age, MS duration, EDSS and MSSS (*R* = 0.59, *p* = 0.00001). A negative correlation was found between P300 amplitude and FSS results but only for Pz reference (*R* = −0.2, *p* = 0.04), without statistical significance for Cz and Fz references.

**Table 5:** Results of a Brief Repeatable Battery of Neuropsychological Tests in non-fatigued (I), moderately fatigued (II) and severely fatigued (III) MS patients

	Subgroup I (*n* = 29)	Subgroup II (*n* = 31)		Subgroup III (*n* = 26)	
SVRT-L					
Mean	35.8	33.9	***p*** **(I–II)** = **0.013**	33.3	*p* (I–III) = 0.054 *p* (II–III) = 0.12
Median	34.0	34.0		34.0	
SD	12.6	13.6		12.9	
SVRT-C					
Mean	27.0	23.7	***p*** **(I–II)** = **0.041**	21.2	***p*** **(I–III)** = **0.003** ***p*** **(II–III)** = **0.037**
Median	27.0	20.0		23.0	
SD	14.9	13.9		12.0	
SVRT-T					
Mean	47.4	44.2	***p*** **(I–II)** = **0.021**	44.0	***p*** **(I–III)** = **0.005** *p* (II–III) = 0.06
Median	48.0	45.0		45.0	
SD	8.2	8.7		8.0	
SVRT-D					
Mean	7.48	6.32	***p*** **(I–II)** = **0.037**	6.65	***p*** **(I–III)** = **0.003** *p* (II–III) = 0.09
Median	8.0	7.0		7.0	
SD	2.71	2.87		2.17	
SpaR					
Mean	19.1	18.0	*p* (I–II) = 0.79	16.7	*p* (I–III) = 0.051 *p* (II–III) = 0.49
Median	18.0	18.0		15.0	
SD	6.8	5.1		5.2	
SpaR-D					
Mean	7.14	8.03	*p* (I–II) = 0.38	5.96	***p*** **(I–III)** = **0.013** *p* (II–III) = 0.14
Median	7.0	6.0		5.0	
SD	2.5	8.65		2.52	
SDMT					
Mean	50.6	46.7	*p* (I–II) = 0.34	38.1	***p*** **(I–III)** = **0.003** *p* (II–III) = 0.48
Median	50.0	48.0		36.0	
SD	14.8	11.9		10.7	
PASAT					
Mean	51.0	46.0	*p* (I–II) = 0.099	43.0	***p*** **(I–III)** = **0.001** *p* (II–III) = 0.073
Median	53.0	49.0		46.5	
SD	9.8	11.8		13.0	
VF					
Mean	25.0	23.8	*p* (I–II) = 0.99	23.6	*p* (I–III) = 0.15 *p* (II–III) = 0.75
Median	26.0	24.0		24.0	
SD	6.3	5.4		5.4	

Bold values indicate *p* < 0.05 which are statistically significant

**Fig. 2:**
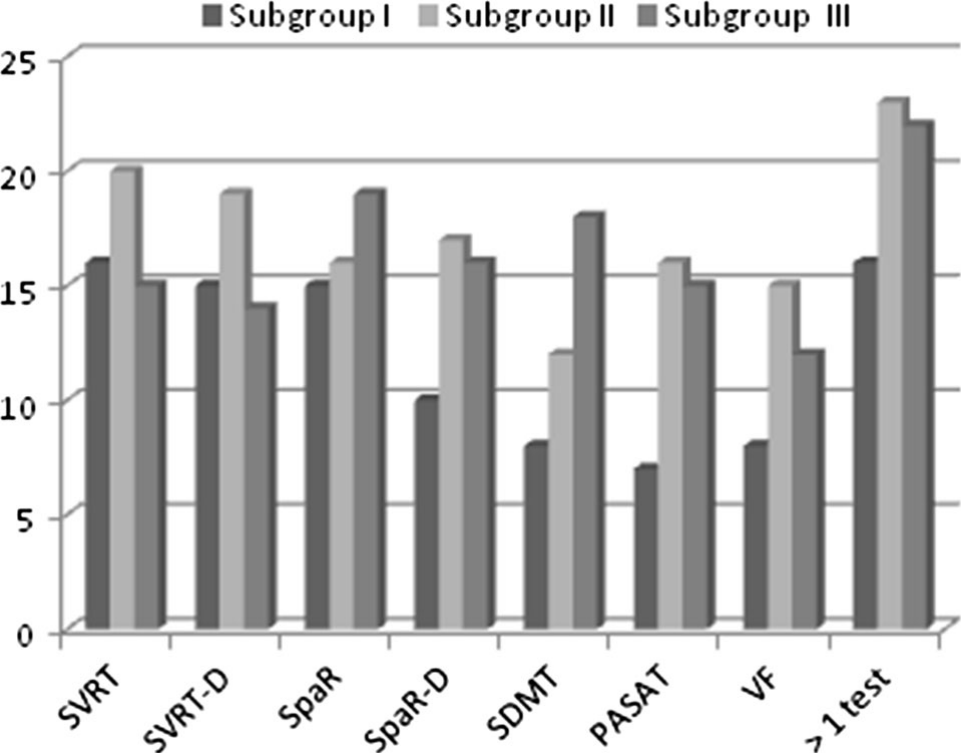
Subjects with abnormal results in Brief Repeatable Battery of Neuropsychological tests compared for the subgroups of non-fatigued (I), moderately fatigued (II) and severely fatigued (III) MS patients

Figure 2 shows the proportion of MS patients from subgroups I, II and III having achieved worse than normative result values in particular tests within BRBNT. Patients in subgroups II and III scored significantly worse than subgroup I in SVRT (Table 6). The delayed results of SpaRT were significantly worse in subgroup III than in I, and for the basic SpaRT, the results were worse but on the border of significance. Patients in subgroup III scored significantly less than those form subgroups I and II in SDMT. The PASAT results were significantly worse in subgroup III than in I and II. No differences between the subgroups were found in terms of VF performance (Table 6).

**Table 6:** Correlations between neuropsychological tests results and fatigue measures, including  multiple regression analysis of correlations between fatigue measures (FSS/FSS-5, MFIS), neuropsychological tests (SDMT, PASAT) and age, disease duration and progression of disability (MSSS) (R–Pearson’s correlation coefficient)

	FSS	FSS-5	MFIS	MFISmod	Physical subscale MFISmod	Cognitive subscale MFISmod
SDMT	*R* = −0.38 *p* = **0.00001**	*R* = −0.38 *p* **=** **0.00001**	*R* = −0.42 *p* = **0.00001**	*R* = −0.42 *p* = **0.0001**	*R* = −0.38 *p* = **0.0003**	*R* = −0.33 *p* = **0.002**
PASAT	*R* = −0.30 ***p*** = **0.004**	*R* = −0.32 *p* **=** **0.005**	*R* = −0.39 *p* = **0.00001**	*R* = −0.33 *p* = **0.002**	*R* = −0.24 *p* = **0.024**	*R* = −0.37 *p* = **0.0005**

SDMT = *f*(FSS, age, disease duration, MSSS) *N* = 86, *R* = 0.53, *R* ^2^ = 0.28, adjusted *R* ^2^ = 0.26, *p* = 0.00000			
	Beta	*B*	*p*
Disease duration	−0.389	−0.730	0.00010
MSSS	−0.439	−2.73	0.00001

SDMT = *f*(MFIS, age, disease duration, MSSS) *N* = 86, *R* = 0.57, *R* ^2^ = 0.32, adjusted *R* ^2^ = 0.30, *p* = 0.00000			
	Beta	*B*	*p*
MFIS	−0.234	−0.198	0.0240
Disease duration	−0.305	−0.572	0.00302
MSSS	−0.358	−2.23	0.00052

PASAT = *f*(FSS, age, disease duration, MSSS) *N* = 86, *R* = 0.30, *R* ^2^ = 0.09, adjusted *R* ^2^ = 0.08, *p* = 0.00442			
	Beta	*B*	*p*
FSS	−0.304	−2.06	0.00442

PASAT = *f*(MFIS, age, disease duration, MSSS) *N* = 86, *R* = 0.39, *R* ^2^ = 0.15, adjusted *R* ^2^ = 0.14, *p* = 0.00022			
	Beta	*B*	*p*
MFIS	−0.388	−0.290	0.00022

Bold values indicate *p* < 0.05 which are statistically significant

Significant correlations were found between the results of SDMT and PASAT and all the fatigue measures: FSS/FSS-5, MFIS, MFISmod, cognitive and the physical subscales of MFISmod (Table 5). Multiple regression analysis showed that among FSS, age, MS duration and MSSS, only MS duration and MSSS were independent factors for SDMT (*R* = 0.53, *R*^2^ = 0.28, adjusted *R*^2^ = 0.26, *p* = 0.00000), while only FSS was independent factor for PASAT (*R* = 0.3, *R*^2^ = 0.09, adjusted *R*^2^ = 0.08, *p* = 0.004) (Table 6). Multiple regression analysis for SDMT and PASAT in relation to the MFIS/MFISmod, age, MS duration and MSSS showed that MFIS/MFISmod, MS duration and MSSS were independent factors for SDMT (*R* = 0.57, *R*^2^ = 0.32, adjusted *R*^2^ = 0.3, *p* = 0.00000), while only MFIS/MFISmod for PASAT (*R* = 0.39, *R*^2^ = 0.15, adjusted *R*^2^ = 0.14, *p* = 0.0002) (Table 6). No other correlations were found between BRBNT results and fatigue measures.

Multiple regression analysis for fatigue measures (FSS) in relation to BRBNT results and ERP parameters showed that SDMT correlated with FSS independently from other BRBNT results and P300 latencies (*R* = 0.38, *R*^2^ = 0.14, adjusted *R*^2^ = 0.13, *p* = 0.00031) and MFIS correlated with SDMT and PASAT independently from other BRBNT results and P300 latencies (*R* = 0.47, *R*^2^ = 0.22, adjusted *R*^2^ = 0.20, *p* = 0.00004).

## Discussion

Analyzing ERP among the whole group of MS patients in comparison with the controls, we found a prolonged latency of both N200 and P300 components and lowered amplitude of the latter. Such abnormalities have already been described in MS patients and associated with impaired cognition, including executive functions, working memory, attention and information processing [6–13]. Further evaluation of ERP with regard to fatigue, revealed a significant increase in N200 and P300 latency only in moderately and severely fatigued patients and a lowered P300 amplitude in those severely fatigued (but only for Cz reference). Moreover, P300 latency correlated significantly with the MFIS result (in both its versions: basic and modified). In the available literature, there are very scarce data on the relationships between ERP parameters and fatigue in MS patients. Nagels et al. [15] suggested that P300 latency might have a prognostic value in treating fatigued MS patients with modafinil: a greater improvement was noted after 4 weeks of treatment in subjects with an initially shorter P300 latency. The authors did not find any correlations between P300 parameters and the level of fatigue, but this was assessed only with the use of the ten-point Visual Analogue Scale. Sandroni et al. [14] found that more severe fatigue reported by patients during ERP performance was associated with prolonged reaction time and also with decreased latency of the P300a component and an increase in the amplitude of both P300a and P300b.

To investigate the link between electrophysiological parameters and fatigue more specifically, we considered the impact of other factors upon ERP results. P300 latency is known to increase with age [7, 8] but a lack of significant age differences between the subgroups I, II and III, as well as the results of multiple regression analysis allowed to eliminate the effect of age. Although ERP results correspond mainly to cognitive performance, some authors suggest they are also influenced by the duration of MS and degree of disability [9, 10]. Indeed, we found that N200 latency (otherwise not related to fatigue measures) correlated with EDSS and tended to correlate with MSSS. In the case of P300 latency, multiple regression analysis showed that its correlation with MFIS/MFISmod results was independent from the impact of all clinical MS-related variables.

Furthermore, to eliminate the influence of sensory deficit in the course of MS upon ERP performance, we deliberately used only the auditory modality, assuming a much greater frequency of visual pathway damage in MS, which is considered also to affect ERP abnormalities [13, 25]. In our previous study [1], analyzing visual and brainstem auditory evoked potentials in the same group of MS patients, significant difference between MS subjects and controls was found only for interlatencies I–III and III–V components of BAEP, with no significant prolongation of the particular components’ latencies in the MS group. Furthermore, only severely fatigued patients showed prolonged latency of V component, which did not correlate with any of the disease-related variables, apart from Brainstem FS. In the current study, lack of significant differences between N100 latencies in subgroups of MS patients and the controls, supports the conclusion that prolonged latencies of N200 and P300 components result from slowed information processing and not from delay in afferent input. Although lowered N100 amplitude was found in MS patients, it occurred both in non-fatigued and severely fatigued ones and only for the single reference. In general, prolongation of P300 latency in our patients seemed to be associated more specifically with fatigue and cognitive performance than with the overall disease burden. Further investigation in the field of relationships between fatigue and cognition might concentrate on early ERP components (N100, N200), considering the role of early stages of modality specific perception and information processing.

The two scales were used for the assessment of fatigue, because (although highly correlated with each other) they were allowed to evaluate its global level as well as its cognitive and physical aspect. P300 parameters correlated significantly with total MFIS and MFISmod results, but surprisingly, the significant correlation was found between P300 latency and MFISmod physical (but not cognitive) subscale score. Many authors claim that subjective worsening of cognitive functioning due to fatigue should not be coalesced with cognitive impairment [2, 3, 5]. The majority of studies have failed to find a relationship between the level of fatigue and the results of neuropsychological tests, even during prospective observation [2, 4, 5, 26–29]. However, some authors [30–32] have suggested that fatigue occurs more frequently in cognitively impaired subjects, both in early and advanced stages of MS, and that performance of repeated neuropsychological tests deteriorates along with increased fatigue reported by the patients. In our material, moderately fatigued MS patients performed worse than non-fatigued ones in tests evaluating verbal memory and those severely fatigued—also in tests on visuospatial abilities, non-verbal memory and attention. The difference between our findings and the above-mentioned studies may be associated with the choice of tools for cognitive function assessment. According to some opinions [4], BRBNT is not sensitive enough to reveal subtle cognitive deficit as well as its relationships with fatigue. Considering the possibility of deterioration in cognitive performance over time due to fatigue, we noticed that severely fatigued patients scored less in delayed parts of SVRT and SpaRT. However, this effect might not necessarily be caused by fatigue but may also be due to impaired long-term memory.

In our study, the results of SDMT and PASAT (mainly evaluating the selectiveness and concentration of attention) correlated with all the fatigue measures; in multiple regression analysis only PASAT proved to be independently linked to both FSS and MFIS. Similar relationships with fatigue measures were shown for PASAT by DeLuca et al. [33] and Heesen et al. [34] and for digit symbol coding—by Andreasen et al. [35]. Functional neuroimaging in fatigued MS patients revealed increased activation of cerebral areas responsible for attention and association during performance of motor activities [36]. Due to some concepts of fatigue, its presence may require greater concentration of attention during motor or cognitive tasks [29]. On the other hand, attention deficit may interfere with the undertaking and performance of some activities, leading to increased fatigue. Links between fatigue and particular cognitive domains (including attention and long-term memory) certainly deserve further investigation.

The multiple regression analysis showed that correlations between SDMT and PASAT and fatigue measures were independent from P300 latencies. Thus, the ERP parameters cannot be treated as direct indicators of the level of fatigue but rather as an electrophysiological marker of cognitive impairment, which seems to be significantly related to fatigue. The fact that parameters of ERP are objective and concise and they reflect global cognitive function (as opposed to the tests evaluating particular cognitive domains), encourages their use in the assessment and monitoring of cognitive performance.

Although cognitive impairment in the course of MS is usually independent from other symptoms and signs of neurological deficit, we aimed to exclude the possible interfering effect of disability and MS duration upon relationships between fatigue and cognitive performance. The multiple regression analysis showed that correlations between SDMT and PASAT results and MFIS/MFISmod scores were independent from the impact of EDSS, MSSS and the duration of the disease. The lack of differences in age and educational level between subgroups I, II and III allowed us also to eliminate these factors as potentially confounding ones.

The limitation of our study is associated with the fact that the assessment of both cognitive performance and fatigue was performed only once, while all the MS symptoms (including fatigue) fluctuate over time. However, our results may serve as a reference point for further future observations, concentrating on particular cognitive domains or selected ERP parameters, as well as considering psycho-affective issues (ex. depression or anxiety) which may affect both fatigue and cognitive performance. Further investigation in this field might provide a better insight in complex relationships between fatigue, cognition and emotional status in MS patients. It is also worth mentioning that our findings concerning fatigue are associated with the natural course of MS (the majority of patients had never been treated with immunomodulatory agents and a small percentage had ceased such treatment at least 6 months before inclusion in the study). This also makes our results a useful reference for the monitoring of the effect of disease-modifying therapies upon various aspects of MS, including fatigue.

In conclusion, we found that fatigue in the MS patients showed significant relationships with cognitive performance (especially within memory and attention domains). Parameters of auditory ERP appeared to be electrophysiological biomarkers of cognitive function, but were not independently linked to fatigue. Both fatigue and cognitive performance should be monitored parallel in the course of MS.
